# The Worst Things in Life are Free: The Role of Free Heme in Sickle Cell Disease

**DOI:** 10.3389/fimmu.2020.561917

**Published:** 2021-01-27

**Authors:** Oluwabukola T. Gbotosho, Maria G. Kapetanaki, Gregory J. Kato

**Affiliations:** ^1^ Division of Hematology-Oncology, Department of Medicine, University of Pittsburgh School of Medicine, Pittsburgh, PA, United States; ^2^ Pittsburgh Heart, Lung, Blood, and Vascular Medicine Institute, Department of Medicine, University of Pittsburgh School of Medicine, Pittsburgh, PA, United States; ^3^ Division of Pulmonary, Allergy and Critical Care Medicine, Department of Medicine, University of Pittsburgh School of Medicine, Pittsburgh, PA, United States

**Keywords:** hemolysis, sickle cell disease, free heme, inflammation, oxidative stress, IL-6, placental growth factor, pulmonary hypertension

## Abstract

Hemolysis is a pathological feature of several diseases of diverse etiology such as hereditary anemias, malaria, and sepsis. A major complication of hemolysis involves the release of large quantities of hemoglobin into the blood circulation and the subsequent generation of harmful metabolites like labile heme. Protective mechanisms like haptoglobin-hemoglobin and hemopexin-heme binding, and heme oxygenase-1 enzymatic degradation of heme limit the toxicity of the hemolysis-related molecules. The capacity of these protective systems is exceeded in hemolytic diseases, resulting in high residual levels of hemolysis products in the circulation, which pose a great oxidative and proinflammatory risk. Sickle cell disease (SCD) features a prominent hemolytic anemia which impacts the phenotypic variability and disease severity. Not only is circulating heme a potent oxidative molecule, but it can act as an erythrocytic danger-associated molecular pattern (eDAMP) molecule which contributes to a proinflammatory state, promoting sickle complications such as vaso-occlusion and acute lung injury. Exposure to extracellular heme in SCD can also augment the expression of placental growth factor (PlGF) and interleukin-6 (IL-6), with important consequences to enthothelin-1 (ET-1) secretion and pulmonary hypertension, and potentially the development of renal and cardiac dysfunction. This review focuses on heme-induced mechanisms that are implicated in disease pathways, mainly in SCD. A special emphasis is given to heme-induced PlGF and IL-6 related mechanisms and their role in SCD disease progression.

## Introduction

**Figure 1:**
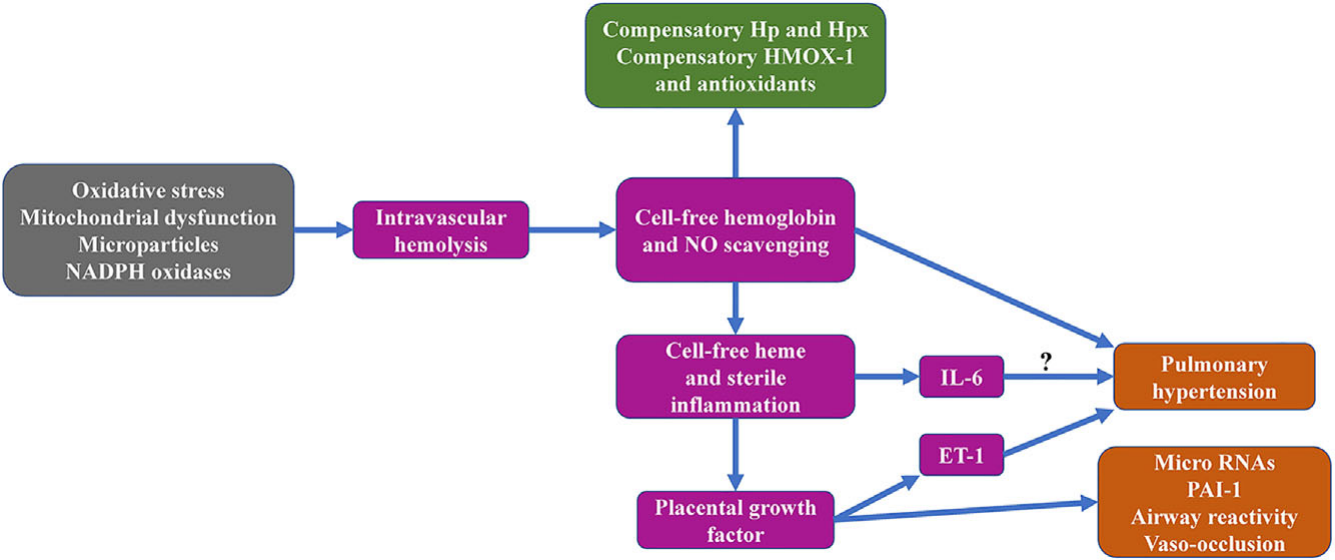
Graphical overview of sickle cell hemolysis-associated topics addressed in the current review manuscript.

Sickle Cell Disease (SCD) is an inherited hematological disorders, with a multi-organ complication affecting millions of people worldwide, especially in sub-Saharan Africa (1). In the United States, there are about 100,000 people with SCD. There are variability and often concurrent complications related to the disease, which may differ in frequency and severity. Accumulating evidence suggests that intravascular hemolysis and hemolysis byproducts including hemoglobin and heme instigate a series of events leading to vascular damage. While hemolysis is a prominent feature of SCD, it is certainly not unique to this disease. Red cell destruction may occur as a result of a hereditary hemolytic disorder, an infection, a medication, cancer, an autoimmune disorder, a cardiomyopathy, a hemorrhagic stroke, trauma or even a blood transfusion, to mention a few (2). The current review focuses on the heme-induced mechanisms that are implicated in disease pathways, mainly in SCD and downstream effects of non-bound (free) heme as a result of intravascular hemolysis caused by sickle cell anemia and other hemolytic disorders (**Figure 1**).

### Heme as a Signaling Molecule in Normal Physiology

Heme synthesis, transport and turnover occurs under normal physiological conditions, and it exerts a physiological signal that helps to control these pathways. For example, heme feeds back to the first committed step in porphyrin synthesis, α-levulinic acid synthase. Heme regulates the Ras-Mitogen Activated Protein Kinase (MAPK) pathway, and it regulates the BACH1 transcriptional repressor, impacting expression of HMOX-1 and β-globin. Heme-regulated inhibitor (HRI) is a eukaryotic initiation factor 2α kinase that coordinates protein synthesis with heme availability in reticulocytes (3). Heme is a crucial prosthetic group for activity of many hemoproteins, include oxygen transport, electron transport, oxygen reduction, and others (4). Heme modulates macrophage differentiation of monocytes to tissue-resident macrophages and stimulates macrophage inflammatory response (5). In sickle cell disease, heme from red cells is turned over *via* both intravascular and extravascular hemolysis pathways that leads to extensive pathology described in the remainder of this review.

## Oxidative Stress and Hemolysis in Sickle Cell Disease

### Reactive Oxygen Species Production in SCD Contributes to Hemolysis

Oxidative stress occurs due to dysregulation between production of reactive oxygen species (ROS) and antioxidants. ROS are vital for cell signaling and homeostasis and are produced as a natural by-product of the normal metabolism of oxygen or exogenously by ionizing radiation and xenobiotic compounds (6–8). Oxidative stress contributes to pathophysiological pathways that underlie inflammation in many hemolytic disorders including SCD (8), β-thalassemia (9, 10), paroxysmal nocturnal hemoglobinuria (11, 12), hereditary spherocytosis (13), and glucose-6-phosphate dehydrogenase deficiency (14–16). RBCs are constantly subjected to oxidative stress due to their role as an oxygen transporter and continuous exposure to both endogenous and exogenous sources of ROS that can damage the RBC and alter blood rheology in SCD patients (17, 18). ROS is generated in SCD through several pathways. Sickle hemoglobin (HbS) produces ROS such as superoxide anion (O2^-^), hydrogen peroxide (H_2_O_2_), peroxynitrite (OONO^-^) and hydroxyl radical (OH.) following auto-oxidation (19). Auto-oxidation is a normal physiological process that generates methemoglobin (metHb, Hb oxidized to Fe^3+^ state with no ability to bind O_2_) and $O_{2}^{-}$ in about 3% of the total Hb every day (19). A small rate of auto-oxidation can produce substantial levels of ROS due to the high concentration of oxygenated Hb (about 5 mM), which can cause enormous damage to the RBC itself, because RBCs make up 40% of the blood volume (20). Moreover, O2^-^ is spontaneously converted to H_2_O_2_ by superoxide dismutase, thereby increasing ROS in the system (19). Excessive amounts of reactive oxygen metabolites is produced due to the unstable nature of HbS resulting in conformational change in the Hb in low O_2_ environment and the continuous auto-oxidation of iron in heme released from Hb (6–8). This heme can oxidize membrane lipids and proteins (21), as evidenced by elevated levels of products of lipid peroxidation including malondialdehyde (MDA) in the plasma of SCD patients (22). Other Hb oxidation products such as ferryl Hb which is also formed in RBCs under conditions of oxidative stress also occurs in HbS (23–25), causing actin remodeling, thereby compromising membrane integrity and transport (26, 27).

### Mitochondrial Dysfunction

The major source of intracellular ROS is the mitochondria in most cells (28) but mature red blood cells (RBCs) from healthy individuals extrude their mitochondria and other organelles during the terminal process of erythropoiesis (29–32). In contrast, a higher percentage of mature RBCs from SCD patients and mice retain their mitochondria leading to excessive ROS accumulation and oxidative stress (25, 33, 34). It has been shown that treatment with products of hemolysis including ferric Hb, ferryl Hb or heme causes bioenergetics changes, abnormal membrane permeability and ROS-induced lipid peroxidation in endothelial and alveolar cells mitochondria (35, 36), which may contribute to inflammatory process and lung injury (37, 38). Additionally, platelets from SCD patients have abnormal mitochondrial activity resulting in oxidant generation and increased activation during vaso-occlusive crisis (VOC) (39). Exposure to cell-free hemoglobin exacerbates this aberrant platelet mitochondrial activity and correlates with markers of hemolysis, NO scavenging and severity of pulmonary arterial hypertension (40).

### Microparticles

Another source of oxidative stress in SCD is erythrocyte-derived submicron membrane vesicles called microparticles (eMPs) (41–44). Plasma eMPs are elevated in sickle cell mice (25), in SCD patients at steady state (41, 44) and during vaso occlusive crisis (45, 46). These eMPs are generated during reoxygenation of sickled erythrocyte (42, 43) or during hemolysis (41, 47). Additionally, thrombospondin-1 (TSP1) may trigger shedding of phosphatidylserine positive eMPs and injection of these eMPs into SCD mice caused vaso occlusion in the kidney (48). These hemoglobin-laden eMPs can transfer heme to endothelial cells, adhere to vascular endothelium and scavenge NO thereby mediating oxidative stress (49–51). Staining of human renal biopsies has been shown to contain hemoglobin-laden eMPs adherent to the capillary endothelium in kidney tissue samples from hyperalbuminuric SCD patients, suggesting that eMPs may contribute to renal injury in SCD (51). Finally, other blood cells such as neutrophils and macrophages also release ROS into the plasma which are neutralized by anti-oxidants such as superoxide dismutase before they can be taken up by RBCs (52).

### Nicotinamide Adenine Dinucleotide Phosphate Oxidases

Vascular smooth muscle and phagocytic cells express nicotinamide adenine dinucleotide phosphate (NADPH) oxidases, which can generate endogenous ROS (53). NADPH oxidase activity is mediated by activation of the small Ras-like GTPase Rac *via* protein kinase C (PKC) stimulation (53). Some plasma factors such as transforming growth factor β1 (TGFβ1) and endothelin-1 (ET-1) have also been shown to stimulate NADPH oxidase activity in neutrophils, monocytes and endothelial cells and many of these factors are present at higher levels in the plasma of SCD patients as a result of persistent inflammatory state associated with SCD (54). RBCs from SCD patients also contain NADPH oxidases, which can generate endogenous ROS, thereby contributing to RBC rigidity and fragility (55).

### Oxidant–Antioxidant Balance

Accumulation of oxidative injury to the erythrocyte distorts membrane integrity, alters blood flow rheology, membrane transport abnormalities, exposure of phosphatidylserine, and cell death (56–58). Despite the numerous pathways by which ROS is generated in SCD, oxidative stress in patients appears to be compensated at steady state, and only becomes deleterious when the balance between ROS production and antioxidants is perturbed due to excessive ROS generation, low antioxidant levels or during crisis (59). Likewise, ROS production becomes markedly amplified in low antioxidant microenvironments, as found in SCD, resulting in damage of macromolecules including lipids (60, 61), DNA (62, 63), and proteins (64, 65).

However, studies of antioxidant levels in SCD patients have yielded variable results, with several studies reporting low (66–69) and others reporting high levels (70, 71) of activity of antioxidant enzymes including glutathione peroxidase (66, 67), superoxide dismutase (67, 70, 72), and catalase (68, 72). These differences may be due to variations in level of disease severity including hemolysis, lipid peroxidation, VOC, acute splenic sequestration and pulmonary hypertension reported in these patients (73–78). Irrespective of the levels detected, the total antioxidant capacity in SCD patients is insufficient to neutralize excess ROS, resulting in oxidative stress (79). Other non-enzymatic antioxidants such as vitamin C and E (80, 81), zinc (76), and selenium (69, 77, 80) are also decreased in SCD patients.

Several approaches to mitigate the harmful effects of oxidative stress in SCD have been proposed such as use of antioxidants (82), neutralization of products of hemolysis with haptoglobin (Hp) and hemopexin (Hpx) (83) and moderate strength and endurance exercise therapy (84). Recent studies showed that increase in physical activity improves blood rheology, increases NO bioavailability and reduction in oxidative stress and hemolysis in mice (85–87) and SCD patients (88).

### Intravascular Hemolysis, Free Hemoglobin, and NO Deficiency

Intravascular and extravascular hemolysis, due in large part to recurrent sickling and unsickling and oxidative stress discussed above, causes premature destruction of RBCs, and contributes to anemia in SCD (56, 89). Rapid production of RBCs ensues to compensate for anemia, resulting in an increased proportion of reticulocytes and younger RBCsin the circulation. Younger RBCs have a higher content of arginase, and with lysis of these younger cells, arginase is released into the plasma during hemolysis (90). This ectopic plasma arginase consumes plasma L-arginine (substrate needed for NO production), and together with consumption of endothelial NO by cell-free plasma Hb contributes to decreased NO bioavailability (91–93). Although consequences of hemolysis in SCD are multifactorial, induction of NO deficiency and oxidative stress by acute and chronic release of products of hemolysis into circulation are major sequelae of hemolysis (94). Depletion of NO promotes a chronic vasculopathy endophenotype that predisposes to pre-capillary pulmonary hypertension, leg ulceration, cerebrovascular arteriopathy, chronic kidney disease and priapism. Details of nitric oxide deficiency and pulmonary hypertension are beyond the scope of this review and have been reviewed in detail elsewhere (94–96).

### Compensatory Mechanisms

Several distinct and overlapping mechanisms have evolved to mitigate the cytotoxic effect of products of hemolysis. Hb dimers are avidly bound by the serum glycoprotein **haptoglobin** (Hp), in the plasma to form Hb-Hp complex, which protects against oxidative damage (97–100). The Hb-Hp complex is recognized and internalized *via* its receptor, CD163, and subsequently cleared by the phagocytic cells in the reticuloendothelial system (97–99). Continuous formation of Hb-Hp complexes in diseases with severe intravascular hemolysis including SCD and paroxysmal nocturnal hemoglobinuria results in depletion of Hp to undetectable levels, leading to some accumulation in plasma of cell-free Hb (101, 102).

### Heme Scavenging Proteins

Cell-free Hb that becomes oxidized or denatured prior to clearance is prone to release free heme. Plasma free heme becomes elevated in SCD patients (103, 104). About 80% of total heme initially binds to plasma lipoproteins including low-density lipoproteins (LDLs) (105, 106) and high-density lipoproteins (HDLs) (107, 108), before being transferred to albumin and Hpx (107, 109). Low levels of these lipoproteins are reported in SCD patients which may be due to increased catabolism or decreased synthesis (110, 111), as low plasma levels also negatively correlated with markers of hemolysis in SCD patients (112–114). Free heme reversibly binds to albumin to form **metalbumin** (115–117), or with high affinity to **hemopexin** (Hpx) (118, 119), and **α1-microglobulin** (120–122).

### Hemopexin

Of all these plasma proteins, Hpx, a plasma glycoprotein produced in the liver has the highest affinity for binding free heme (118, 119, 123), resulting in the formation of Hpx-heme complexes that are removed by endocytosis *via* the Hpx receptor (CD91) in hepatocytes and macrophages (124, 125). After delivering heme to CD91-expressing cells for internalization and degradation by heme oxygenase 1 (HMOX-1), at least some of the Hpx molecules can be recycled back into plasma. Elevated eMPs also correlated with increase in hemolysis markers and low Hpx in SCD patients (126). In the same patients cohort, high eMPs positively correlated with elevated TRV, linking Hpx depletion to increased eMPs and hemolysis, which may predispose patients to pulmonary hypertension (126). In another study, low Hpx negatively correlated with lipid oxidation in human and mice with SCD, with postmortem analysis in SCD patients showing oxidized LDL deposits in the pulmonary artery (127). These reports showed that delayed clearance of heme in circulation due to low plasma Hpx may activate deleterious downstream pathological pathways that may contribute to morbidity and mortality in SCD patients.

### Heme Oxygenase-1

HMOX-1 is an evolutionarily conserved and rate limiting enzyme that degrades heme into equimolar amount of iron, biliverdin and carbon monoxide (108, 128, 129). HMOX-1 is highly expressed in human and mice with SCD and further upregulated on exposure to heme (130, 131). Heme-induced oxidative stress exceeds the capacity of HMOX-1 to prevent cellular and organ injury in transgenic murine model of SCD. Augmentation of HMOX-1 level and activity *via* gene transfer approaches, or pharmacological activation through NRF2 (132), the transcription factor that regulates HMOX-1 expression, conferred protection from heme-induced lung injury (133), vaso-occlusion (134), liver injury (135), kidney injury (136), erythrocyte membrane damage (137), endothelium activation and adherence (135), activation of immune cells and production of inflammatory cytokines (138). Still, the effect of NRF2 activation on hemolysis, γ-globin levels or stress erythropoiesis in mouse model of SCD is controversial (136–138). Not all heme and Hb are bound to proteins or other macromolecules. Unbound heme or hemoglobin in circulation causes erythrocyte membrane damage and injury, activates proinflammatory signaling pathways in RBCs, immune and endothelial cells, hepatocytes, macrophages and neutrophils (105, 139).

### Antioxidant Enzymes

Heme induces a program of antioxidant enzymes that compensate for its intrinsic oxidant stress. These include glutathione S-transferase pi (GSTpi) and NAD(P)H dehydrogenase [quinone] 1 (NQO1) (140).

## Heme and Sterile Inflammation in Sickle Cell Disease

Hemolysis is a major driver of sterile inflammation in pathological conditions including SCD (94, 103, 141), malaria (142, 143), sepsis (144, 145), and also a marker of severity and survival in these patients (146–149). Following hemolysis, Hb is oxidized to unstable methemoglobin resulting in release of free heme (139), which can intercalate into cell membrane and alter cellular structures or taken up by cells (150, 151).

### Intravascular Hemolysis Releases Cell-Free Heme

Free heme accumulates in the plasma in both acute and chronic hemolysis when the rate of intravascular hemolysis exceeds the capacity of circulating heme-binding proteins (152), including Hp and Hpx, which are depleted in human and mice with SCD patients (59, 104, 114, 126, 127, 153–156). There is an emerging concept of small molecular weight scavenging protein such as α1-microglobulin, becoming the predominant heme scavenger when plasma Hpx is low (59). Binding of free heme to different scavenger impacts clinical manifestation of excess heme in circulation as heme-Hpx is trafficked to and recycled primarily in the liver while heme-bound α1-microglobulin are taken to the kidney (59). This phenomenon was demonstrated in a recent publication from Ofori-Acquah and colleagues. They showed that hemopexin deficiency correlates with a compensatory increase in α1-microglobulin in both human and mice with SCD (155). Elevated α1-microglobulin and low hemopexin was also associated with increase in acute kidney injury biomarkers urinary KIM-1 and serum NGAL in SCD patients. The authors showed that this heme-bound α1-microglobulin is directed to the kidney for clearance resulting in acute kidney injury in sickle cell mice (155). Also, acute kidney injury may occur *via* complement deposition in the kidney during intravascular hemolysis and in Hpx deficient condition in SCD mice (157). Patients with SCD with higher plasma levels of free heme also have greater frequency of VOC and acute chest syndrome (158). Accumulation of free heme in plasma is not only cytotoxic, but also mediates generation of free radicals *via* the Fenton pathway (159–161).

### Detection of Heme and Hemoglobin

Assay of cell-free heme and Hb may be an important tool for diagnosis in disease conditions characterized by hemolysis (152, 162). Accurate quantification of heme species may result in early therapeutic intervention before irreversible damage to organs occurs. Currently, most commercially available assays measure total heme (free heme and heme bound to proteins) and are not specific for measuring cell-free heme or Hb. There is a possibility of overestimating or underestimating these heme species. Moreover, free heme is likely a more potent mediator of organ injury and signal transductions, its accurate quantification as a biomarker in disease conditions may be vital. Researchers have developed detection methods using the spectral deconvolution method, antibody capture ELISA or western blotting, reversed‐high‐performance liquid chromatography, and fluorescence-based assays to measure Hb and CFH (103, 152, 162–165). Although these are not commercially available currently, they present an opportunity to quantify different heme species in relation to pathogenesis and therapeutic efficacy in hemolytic conditions.

### Cell-Free Heme in Inflammation

Free heme can induce inflammation *via* direct activation of RBCs (166, 167), macrophages (168–170), neutrophils (171), and endothelial cells (139, 172–174) to secret proinflammatory cytokines including toll-like receptors (TLRs), tumor necrosis factor (TNF), interleukin-6 (IL-6), placenta growth factor (PlGF), interleukin 1 beta (IL-1β) (105, 139, 169, 175, 176) and release of erythroid damage-associated molecular patterns (eDAMPs) that potentiates inflammation (177, 178). Heme has been shown to induce production of IL-1β by activated monocytes/macrophages, endothelial and smooth muscle cells through a nucleotide-binding domain and leucine-rich repeat-containing protein 3 (NLRP3) inflammasome dependent mechanism (139, 169, 172). High mobility group box 1 (HMGB1), a nuclear protein released during systemic inflammatory response, has also been shown to mediate ROS-dependent activation of endothelial cells to secrete IL-1β *via* NLRP3 activation (179, 180). Elevated circulating HMGB1 is associated with inflammation in hemolytic disorders including SCD and sepsis (181–184), suggesting a shared inflammatory signaling pathway through TLR4/Bruton tyrosine kinase for both heme and HMGB1 in SCD (185, 186). Heme can also directly affect the vasculature in mice, as recently shown with loss of heme exporter, feline leukemia virus subgroup C receptor 1a (FLVCR1a) in endothelial cells resulted in disruption of microvessel architecture (187).

### Cell Adhesion Pathways

Cell-free heme also contributes to inflammation by activating cell adhesion pathways. This includes activation of adhesion molecules such as vascular cell adhesion molecule-1 (VCAM-1), intercellular adhesion molecule 1 (ICAM-1), selectins (L, P and E), all involved in mediating cell adhesion to the vascular endothelium *via* activation of integrin αMβ2 on neutrophils (188–192). Besides, several studies in the last decade have associated hemolysis and selectins expression with RBCs adhesion to endothelial cells (193–195), acute lung injury (196), vaso occlusion (197), pain (198, 199), liver injury (200–202), and kidney injury in SCD (83).

P-selectin is associated with platelet-neutrophil aggregate formation that contributes to inflammation, pulmonary dysfunction and lung vaso occlusion in SCD (200, 203). In addition, a recent study by Merle and colleagues, showed a direct link between heme-induced TLR4 and complement system activation on liver endothelium mediated by P-selectin, with genetic or pharmacological blockade of P-selectin or complement system ameliorating liver injury in mice (202). This expansive body of works culminated in clinical trial and eventual FDA approval of P-selectin blockade therapy for the prevention of pain crises in SCD (198, 199). Furthermore, persistent inducibility of endothelium-derived adhesion molecules by proinflammatory cytokines such as TNF-α and IL-6 coupled with chronic hemolysis in SCD patients ultimately results in VOC, organ dysfunction and early mortality (101, 204–208). There are several ongoing clinical trials in SCD looking at mediating the effect of inflammation-induced organ damage *via* some of the mechanisms discussed above.

### Hemolysis, Inflammation, and microRNAs

Recent evidence supports a potential role of microRNAs (miRNAs) in complications of SCD (209, 210) and malaria (211, 212), both pathological conditions with hemolysis, suggesting a role for heme modulation of miRNAs. miRNAs are noncoding RNAs of 22 nucleotides in length that regulate the expression of their target genes post-transcriptionally (213). miRNAs are involved in important biological processes including apoptosis (214), hematopoietic differentiation (215) and cell proliferation (216). miRNAs are important regulatory molecules and activation of immune response during initiation and progression of many diseases inflammatory diseases such as cancer, Crohn’s disease, rheumatoid arthritis, systemic lupus erythematosus, and asthma, *via* expression of proinflammatory cytokines including TNF-α and TLRs (217–222). There are studies linking heme and miRNAs processing in mammalian cells. Heme binds directly to the RNA-binding protein DiGeorge critical region-8 (DGCR8), which is essential for the first miRNA processing step (213, 223–225). Hemolysis elevates the expression of several miRNAs found in RBCs including miR-16, miR-92a, miR-451, and miR-486 (226, 227). There is upregulation of some miRNAs including miR-16, miR-451 and miR-144 in reticulocytes from SCD patients (228, 229). Conversely, elevated levels of these miRNAs also correlated with severe anemia, increased sensitivity to oxidative stress, downregulation of NRF2 and decreased intracellular glutathione levels (230, 231). On the other hand, members of the miR-154, the miR-329 and miR-376 family, involved in TGF-β signaling pathway are downregulated in platelets of SCD patients (210). Although few numbers of studies have reported the involvement of miRNAs in complications of SCD (232), however, there is a gap in knowledge of how stress or heme regulation of these miRNAs and exposure of immune cells to proinflammatory cytokines that are elevated in SCD might play a role in organ dysfunction. Targeting these miRNAs in SCD might offer novel therapeutic strategy in preventing hemolysis-induced inflammation and end organ damage, especially in the heart, lung, liver, and kidney where miRNAs are abundant (222, 233–240).

## Hemolysis and Organ Damage in Sickle Cell Disease

SCD patients on average live longer today than 50 years ago. This is due to progress in understanding the mechanisms and risk factors of several complications of the disease, associated clinical findings and mouse models, approval of new treatment therapies, multi-disciplinary approach to care, penicillin prophylaxis and high-tech diagnostic tools (241). However, this reduction in childhood mortality gives rise to an older population of patients that develop age-related chronic organ damage, driven in part by hemolysis (94). Hemolysis-induced extensive and sometimes irreversible organ damage continues to be a major source of morbidity and mortality in SCD. Even transplanted organs are also at risk of failure in SCD patients due to hemolysis and sickling (242). Therefore, there is a need for research to understand the fundamental mechanisms involved in heme-mediated organ damage in SCD patients. Over the years, several studies in the general population as well as in SCD suggest that hemolysis causes injury to the kidney (243–245), lung (246), heart, and liver. We have summarized some of the impacts of hemolysis on different organs in **Table 1**.

**Table 1:** Summary of current literature supporting a damaging role of hemolysis in different organs.

Organ	Impact of heme damage	References	Disease/model
Kidney	Proximal tubule dysfunction and impaired vitamin D metabolism	(247, 248)	Cell culture/mice
	Proteinuria, acute and chronic injury, and iron deposition	(244, 245, 249–253)	Human
	Acute renal failure, oxidative stress, inflammation, and toxicity	(254–257)	Human/mice
	Acute renal vasoconstriction *via* TLR4 signaling	(258, 259)	Cell culture/Mice
	Apoptosis in proximal tubular epithelial cells *via* caspase-dependent/-independent pathways	(260, 261)	Cell culture
	Endothelial apoptosis and vaso occlusion	(262)	Human/cell culture/mice
Lung	Acute chest syndrome *via* TLR4, NRF2 and p-selectin signaling	(133, 196, 263)	Cell culture/mice
	Oxidative injury and progression of pulmonary hypertension (PH)	(262)	Cell culture/mice
	Angioproliferative PH *via* accelerated purine metabolism	(264)	Rats
	Acute lung injury *via* increased alveolar capillary barrier dysfunction	(265, 266)	Human/cell culture/mice
	Oxidation and mitochondrial dysfunction in epithelial lung cells	(36)	Cell culture
Liver	Increased vascular ICAM-1 expression on blood vessels and vaso occlusion	(267)	Cell culture/mice
	Advanced fibrosis and iron overload	(268)	
	Oxidative stress, neutrophil infiltration, and extravasation through NF-κB activation	(269)	
Heart	Impaired nitric oxide bioavailability and pulmonary hypertension	(270, 271)	Mice
	Smooth muscle proliferation *via* NADPH oxidase activity, atherosclerosis, and hypertension	(101, 272)	Cell culture
	Increased risk of cardiovascular disease	(273, 274)	Human
	Endothelial activation and altered cardiac function	(275, 276)	Mice
	Mitochondria dysfunction	(277)	Human/cell line
	Ischemic injury	(278)	Human/cell culture/mice
	Contractile dysfunction due to altered contractile proteins	(279)	Human primary cardiomyocytes

## Placenta Growth Factor

In addition to its role as a DAMP, heme promotes the expression and secretion of placenta growth factor (PlGF), a pleiotropic growth factor already known to influence multiple pathways contributing to the pathophysiology of SCD (167, 176, 280). PlGF is a member of the Vascular Endothelial Growth Factor (VEGF) family. It was originally cloned from a human placenta cDNA library in 1991 (281), hence the name, but since then it has been detected in a wide variety of tissues (282). PlGF has a partial sequence similarity to VEGF-A but the two molecules share a remarkable topological identity (283). There are four human isoforms (PlGF 1–4), which are generated by alternative splicing and are slightly different in size. PlGF-1 (131 aa) and PlGF-2 (152 aa) are the predominant isoforms in humans. On the contrary, mice carry a single isoform, PlGF-2 (140 aa).

PlGF exists as a homodimer or as a heterodimer with VEGF. PlGF is a ligand for the transmembrane and soluble form of the vascular endothelial growth factor receptor 1 (VEGFR-1, Flt-1) (284), which can also bind VEGF. Distinct from VEGF, PlGF does not bind vascular endothelial growth factor receptor 2 (VEGFR-2, Flk-1) but it can affect VEGFR-2 signaling in an indirect manner (285–287). PlGF-2 can also bind heparin and the transmembrane neuropilin receptors 1 and 2 (NRP1 and NRP2) (288, 289). In addition to its role as a receptor binding competitor of VEGF (284), PlGF can exert its own biological effect upon binding to VEGFR-1. Depending on the cell type, PlGF binding upregulates VEGF, fibroblast growth factor 2 (FGF2), platelet derived growth factor beta (PDGFB) and matrix metalloproteases (MMPs) (290, 291). Furthermore, PlGF receptor binding is shown to activate an intermolecular crosstalk regulator between VEGFR-1 and VEGFR-2, often resulting in enhancing VEGF/VEGFR-2 signaling (287). It is important to emphasize here that PlGF or VEGF binding to FLT1 results in discernible receptor phosphorylation patterns and induction of distinct signaling pathways (287, 292, 293). PlGF expression is induced by hypoxia, probably in a cell specific manner, but the exact mechanism remains elusive in the absence of hypoxia responsive elements (HRE) at the gene’s promoter region (294, 295). So far, the association of only a few transcription factors has been verified for the PlGF promoter: metal transcription factor 1 (MTF-1) (295), NF-kB (296), forkhead box D1 (FoxD) (297), erythroid Kruppel-like factor (EKLF) (167), nuclear factor erythroid 2 like 2 (NRF2) (176), glial cell missing 1 (GCM1) (298). Posttrascriptional regulation of PlGF has also been reported through the regulation of the protein kinase C (PKC), p38 mitogen activated protein kinases (p38 MAPK), c-jun N-terminal kinase (JNK) and Ras-dependent extracellular signal-regulated kinase 1/2 (ERK1/2) signaling pathways (299, 300).

Surprisingly, PlGF seems to have a redundant role under normal conditions (285) but becomes very important in disease situations, where fluctuations of its levels cause a variety of issues in multiple biological processes. Because of that reason, PlGF-based therapeutic approaches have been proposed as disease specific with minimal impact for healthy cells (301). The most well established role of PlGF is in angiogenesis and more specifically in neo-angiogenesis in pathological conditions such as ischemia or cancer (285, 302, 303). PlGF’s pleiotropic nature in evident in its angiogenic role where it exerts a paracrine or autocrine effect on endothelial cells, smooth-muscle cells, fibroblasts, bone marrow progenitor cells and monocytes, to orchestrate vessel growth and maturation (304). The description of the full spectrum of PlGF’s biological role is beyond the scope of this review but to mention a few, PlGF plays a role in inflammatory response (305, 306), promotes bone repair (307), sustains the proangiogenic M2 phenotype of tumor associated macrophages (308), affects dendritic cell differentiation and maturation (309), supports the generation of an inflammatory status driving adaptive cardiac remodeling (310). To summarize, all the evidence to date supports a role for PlGF in pathogenic angiogenesis and inflammation well outside the realm of pregnancy. Through mitogen and migratory effects on endothelial cells as well as macrophage activation and chemoattraction, PlGF emerges as a driver and marker of a plethora of seemingly diverse pathologies, especially angiogenesis and inflammation.

## Hemolysis, PLGF, and Complications of Sickle Cell Disease

**Figure 2:**
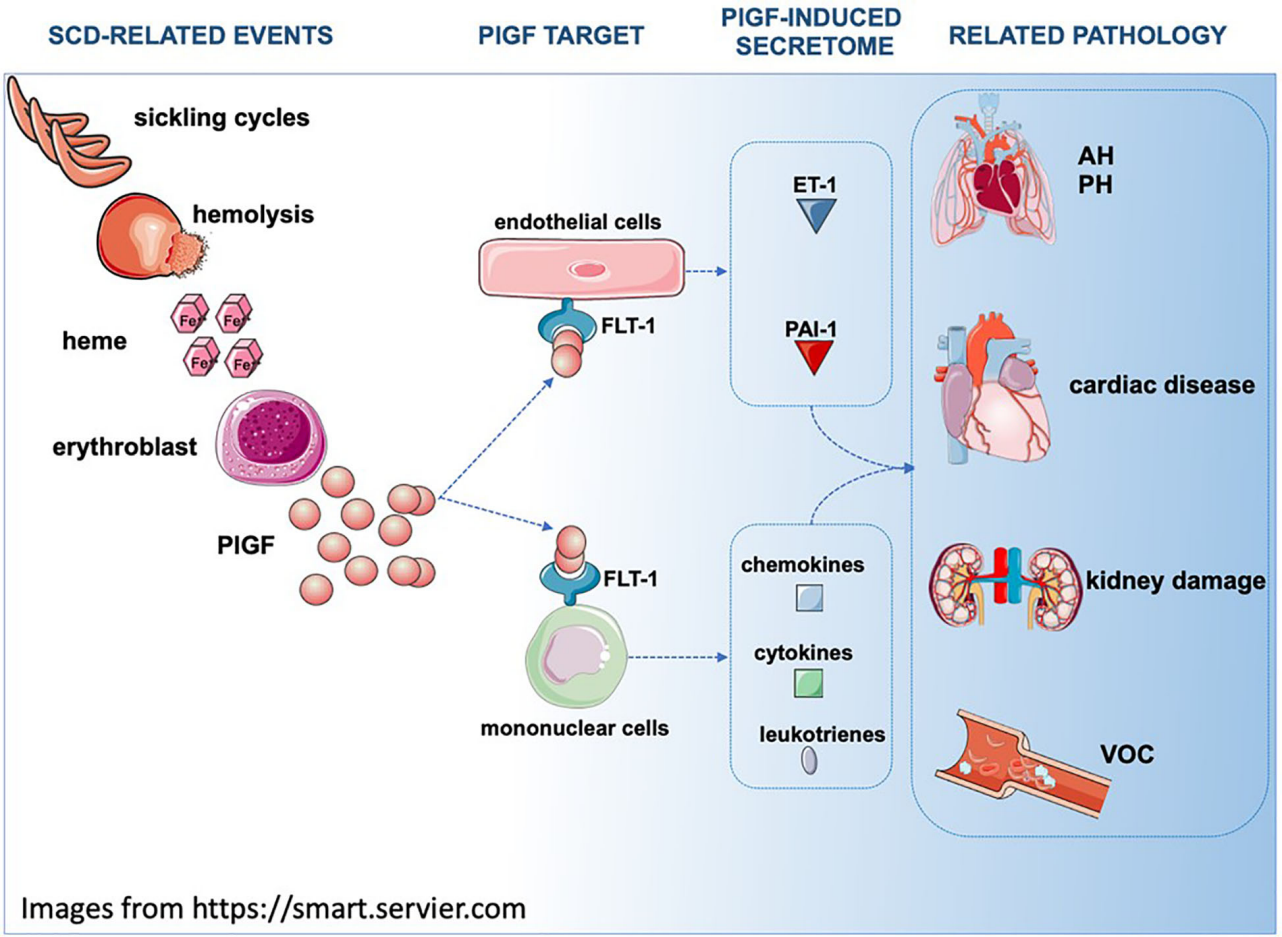
In SCD, repeating sickling cycles result in increased hemolysis. Hemolysis byproducts such as heme induce PlGF expression in multiple cell types (for simplicity purposes only erythroblasts are depicted). Secreted PlGF is a ligand for FLT-1 receptor and triggers the expression of ET-1, PAI-1, leukotrienes and cytochemokines, affecting the physiology of multiple organs. AH, Airway hyperreactivity; PH, Pulmonary hypertension; FLT1/VEGFR1, Fms related receptor tyrosine kinase 1; PlGF, placenta growth factor; ET-1, endothelin 1; PAI-1/Serpine1, plasminogen activator inhibitor 1.

One of the least appreciated roles of PlGF is the one that it has in hematopoiesis (311, 312) and in hemoglobinopathies (313) (**Figure 2**). Plasma PlGF is elevated in SCD patients and the increase correlates with the severity of hemolysis, endothelin 1 (ET-1) expression, the occurrence of pulmonary hypertension (167, 280, 314, 315) and VOC (316, 317).

### Pulmonary Hypertension

PH is a serious complication in sickle cell patients, which is associated with high mortality (318). A variety of biological pathways and disease related pathologies contribute to the development of PH and many of them involve free heme and upregulation of PlGF. Along with PlGF, ET-1, a potent vasoconstrictor, is significantly higher in the blood of sickle patients (167, 316, 319, 320) suggesting a mechanistic link between the two factors. In support of this connection, the overexpression of PlGF in healthy mice using lentiviral gene transfer results in increased ET-1, increased right ventricle pressure and right ventricle hypertrophy as early as 8 weeks after PlGF gene transfer (280). In vitro PlGF stimulation of cultured human pulmonary microvascular endothelial cells (HPMVEC) revealed that ET-1 induction was mediated by PI-3 Kinase, NADPH-oxidase, and HIF-1a (314). Interestingly, HIF-1a stimulation of the ET-1 promoter is hypoxia independent and occurs upon the direct binding of HIF-1a on the HRE elements of the ET-1 promoter. In a similar manner, PlGF upregulates endothelin-B receptor (ET-BR) in monocytes, priming them to be over-stimulated by ET-1 and produce higher levels of chemokines MCP-1 and IL-8 (314). Both MCP-1 and IL-8 are elevated in SCD patients (321) supporting the PlGF-ET-1 synergy as another contributing factor to the development of PH in SCD.

### Regulation of miRNAs

On a post-transcriptional level, PlGF attenuates miR-648 and miR-454, which recognize and bind the 3’ UTR of ET-1 mRNA. The association of low miR-648/miR-454 with high ET-1 and PlGF levels is supported in both *in vivo* and *in vitro* studies (322, 323). Furthermore, PlGF attenuates miR-199-5p, which binds the 3’UTR of HIF-1a mRNA, creating another level of control over ET-1 expression (324). The molecular repression of miR-199-5p by PlGF is mediated by the upregulation of the activating transcription factor 3 (ATF3) which upon binding causes deacetylation and chromatin condensation at the miR-199-5p locus (325). Similar to miR-648, the association of low miR-199-5p levels with high PlGF and ET-1 levels is supported by *in vivo* and *in vitro* studies (324).

### Plasminogen Activator Inhibitor 1

PlGF is also linked to the increase in PAI-1 levels in the plasma and lungs of sickle cell patients and humanized sickle mice respectively (326). PAI-1 is increased during steady state SCD but its expression is exacerbated during VOC. Elevation of PAI-1 levels is associated with decreased fibrinolytic capacity (327) and is believed to contribute to the SCD prothrombotic state and the development of PH (328). In vitro PlGF stimulation induced PAI-1 expression in pulmonary microvascular endothelial cells and monocytes through the activation of c-jun N-terminal kinase (JNK), hypoxia inducible factor 1a (HIF-1a) and nicotinamide adenine dinucleotide phosphate (NADPH) oxidase (326). In addition, PlGF expression affects the stability of PAI-1 mRNA by downregulating microRNAs miR-454, miR-301a, and miR-30c which recognize and bind the PAI-1 3’-UTR. PlGF regulation of miR-454 and miR-301 is mediated by PPARa and HIF-1a (323). All of these microRNAs are detected in significantly lower levels in SCD patients compared to healthy controls (323, 329). In vivo experiments using PlGF null and SS sickle mice as well as adenoviral overexpression of PlGF, have confirmed that PlGF plays a significant role in PAI-1 regulation (326).

### Inflammation and Airway Hyper-Reactivity

Airway hyper-reactivity is a common complication in SCD, especially in younger patients (330), and correlates with biomarkers of hemolysis (331). Patients show elevated levels of circulating leukotrienes (332) and their monocytes express higher levels of 5-lipoxygenase (5-LO) and 5-lipoxygenase activating protein (FLAP), both involved in leukotriene synthesis (333). Consistent to its proinflammatory nature, PlGF induces leukotriene production which in turn increases inflammation and airway hyper-reactivity, both key features of SCD. As in the case of PAI-1, the induction is mediated by HIF-1a and NADPH oxidase (333). Further studies have confirmed PlGF as an important regulator of leukotriene production and airway hyperactivity in SCD and asthma (332).

### Vaso-Occlusion

Activated leukocytes in sickle cell patients are considered a significant promoting factor for VOC (334). Activated mononuclear cells from SCD patients express high levels of the cytochemokines VEGF, IL-1β, monocyte chemotactic protein 1 (MCP-1), IL-8 and macrophage inflammatory protein-1 beta (MIP-1β). In vitro studies have shown that monocytes from healthy individuals can be activated by PlGF to increase the expression of proinflammatory cytokines and chemokines such as TNF-α, IL-1β, MCP-1, IL-8, and MIP-1β (316, 335). This activation is achieved by the PlGF-VEGFR-1 interaction and involves the PI-3 kinase/AKT and ERK-1/2 signaling pathways (335). Because VOC in SCD is promoted by inflammation and leukocyte adhesion stimulated by cytokines (197, 336, 337), antibody neutralization of PlGF was tried successfully for reduction of inflammation and vaso-occlusive complications in murine SCD models (317). Regulation of PlGF levels could also be achieved by manipulating factors that control its transcriptional or translational expression. Per instance, pharmacological upregulation of miR-214 which is known to bind PlGF 3’-UTR, could be engaged to reduce PlGF levels (338).

### Renal Dysfunction

PlGF is significantly upregulated in the serum of patients with chronic kidney disease and decreased renal function, supporting a potential mechanistic link between PlGF and kidney function (339, 340). Sickle cell nephropathy (SCN) is an complex phenotype which encompasses almost every physiological process in the kidney, leading to complications that may range from common and relatively mild to rare and life-limiting (243). In SCD patients markers of renal dysfunction are associated with elevated ET-1 serum levels (341) and studies in sickle cell mice have shown that ET-1 can cause renal injury, likely mediated by ROS (342). Although it has not been shown experimentally, sickle cell-related elevated PlGF levels could possibly contribute to higher ET-1 levels (167, 314) driving renal dysfunction. However, administration of exogenous heme in control and sickle cell mice has been shown to result in the upregulation of PlGF in the murine kidneys in agreement with heme uptake from renal cells and HMOX-1induction (343). In addition to ET-1, PAI-1 has also been shown to play a role in nephropathies (344) but its role in SCD or its potential regulation by PlGF remains unexplored.

### Cardiac Dysfunction

Cardiac complications are common in SCD patients and along with the pulmonary complications raise their morbidity and mortality risk (94, 345). There has been accumulating evidence that PlGF dysregulation is present in multiple heart conditions although it is often unclear if it is only a disease biomarker or it actively promotes disease pathogenesis. In patients with chronic kidney disease, PlGF levels are associated with higher incidence of cardiovascular events and mortality (340). In the same disease, PlGF is an independent risk predictor for left ventricular diastolic dysfunction (346). In human atherosclerotic plaques, the expression of PlGF is associated with plaque destabilization and disease manifestation (347). The pro-atherosclerotic role of PlGF is corroborated in rabbits where PlGF adenoviral expression promotes atherogenic intimal thickening and macrophage accumulation in the carotid artery (348). PlGF is also elevated in the plasma of patients with acute coronary syndromes where it can be used as a risk predicting biomarker (349). PlGF promotes cardiac hypertrophy *via* endothelial cell release of NO which induces cardiomyocyte growth (350) and by inducing the secretion of paracrine factors (IL-6, IL-1b, Cxcl1) from endothelia and fibroblasts that promote cardiac adaptation and hypertrophy (351–353). In the case of ischemic cardiomyopathy, PlGF has been reported both as promoting the disease (354) and as a potential therapeutic (355). The apparent controversy could be due to differences between a local and acute administration of an angiogenic factor (355) compared to a more systemic and chronic upregulation (354). Our research has shown that PlGF is elevated in the hearts of sickle mice and it is further induced after administering exogenous heme (343). Surprisingly, the level of PlGF induction is comparable to that of the liver which is considered the major heme detoxifying organ (343). An interesting finding of this study is that mouse hearts have high levels of HMOX-1, which are further increased by heme induction, and that they show no heme accumulation unless NRF2 is depleted. These data suggest that cardiac tissue has the ability to detoxify heme *via* the NRF2 antioxidant response pathway.

## Hemolysis, Interleukin-6, and Cardiovascular Dysfunction

IL-6 is a ubiquitous and pleiotropic proinflammatory cytokine produced by many cells including macrophages (356, 357), neutrophils (358, 359), endothelial and smooth muscle cells (360, 361), cardiomyocytes (362) and fibroblasts (363), when stimulated by ligands for toll-like receptors or other pattern recognition receptors. IL-6 is a glycoprotein composed of 184 amino acids and of 26 kDa in molecular weight (364). Currently, there are ten cytokines belonging to the IL-6 family; IL-6, IL-11, ciliary neurotrophic factor (CNTF), leukemia inhibitory factor (LIF), oncostatin M (OSM), cardiotropin-1 (CT-1), cardiotrophin-like cytokine (CLC), IL-27, neuropoietin (NP), and IL-31 (365). IL-6 regulates many biological functions including hematopoiesis (366), oncogenesis (367) and differentiation of B cells (368), induction of acute phase proteins and immune regulation (369). Additionally, IL-6 plays a vital role in chronic inflammatory processes in various cells and disease conditions (364). IL-6 signaling is through two pathways; classic/cis-mediated signaling *via* membrane-bound IL-6 receptor (mIL-6R) or trans-mediated signaling *via* the soluble form of IL-6R (sIL-6R) (364, 369). Classic/cis-signaling occurs in cells that express IL-6R such as hepatocytes, neutrophils and monocytes (365, 369). Conversely, trans-mediated signaling occurs after secretion of sIL-6R by RNA alternative splicing, ectodomain shedding or proteolytic cleavage of mIL-6R (370), which in turn stimulate cells (365, 369). Once IL-6 binds to mIL-6R or sIL-6R, the cytokine forms a complex with the ubiquitously expressed membrane protein gp130, a shared signal-transducing receptor of all IL-6 type cytokines (370). Dimerization of the receptor complex activates Janus kinases (JAKs) resulting in phosphorylation of the tyrosine residues in the cytoplasmic domain of gp130 (364, 371). Activation of JAKs triggers the extracellular-signal-regulated kinase (ERK), mitogen-activated protein kinase (MAPK) and signal transducer and activator of transcription (STAT) signaling pathways (370, 371). However, IL-6 role in pathophysiology of chronic inflammation and diseases is driven *via* IL-6 trans-signaling because classic/cis-signaling *via* the mIL-6R is limited to few cells that express IL-6R (372). Blockade of IL-6 trans-signaling is effective in attenuating proinflammatory activities of IL-6 in several disease conditions (365).

Several studies in human and rodents found hemolysis and elevated IL-6 occurring concurrently. Hemolysis and elevated IL-6 are associated with disease severity in malaria (373, 374), sepsis (375) and pre-eclampsia (376), with cardiac dysfunction as an additional comorbidity in these diseases. Besides, elevated cardiac IL-6 is also associated with cardiac hypertrophy and fibrosis in the general population (362, 377) and in rodents (378, 379). In malaria, elevated IL-6 is found in patients with severe *Plasmodium falciparum/vivax* malaria and associated with development of cardiac complications (373, 374). Sepsis patients with elevated IL-6 are at a higher risk of developing cardiac dysfunction which may be due to direct negative inotropic effect of IL-6 mediated *via* altered production of myocardial nitric oxide (375), altered calcium homeostasis (380, 381) and impaired β-adrenergic signaling (382–384). Elevated IL-6 in pre-eclampsia patients result in reduced anti-inflammatory protection in the maternal vascular system (385) and stimulation of vasoactive substances including angiotensin II type 1 receptor and endothelin-1 (386). Although, elevated plasma IL-6 have been reported in human and mice with SCD (168, 387, 388), and hemolysis is a major comorbidity of SCD (94), however, there has been no direct link between these two processes. Conversely, left ventricular hypertrophy (LVH) is found in over 60% of children and 37% in adults with SCD (389, 390), with cardiopulmonary complications accounting for about 26% of deaths in adults with SCD (391). In this current issue and for the first time, our group investigated the expression of plasma and cardiac IL-6 and its inducibility by heme in Townes sickle cell (SS) mouse model (392). We observed significantly elevated cardiac IL-6 and direct heme induction of circulating and cardiac IL-6 transcripts and protein in SS mice compared to controls. We showed that this heme-induced IL-6 is NRF2-independent in the heart. Our results of heme-induced IL-6 is in agreement with elevated levels of IL-6 reported in cardiac cells treated with Hpx and in heart isolated from Hpx deficient mice (393). Because our data showed upregulation of cardiac hypertrophy genes following heme treatment in SS mice, there is a possibility that heme is inducing IL-6 in the heart and prolonged activation and exposure to IL-6 could contribute to LVH in SCD patients. We are currently investigating potential mechanism(s) and specific cell-types secreting IL-6 in the heart of SS mice. There are several pathways through which heme may induce IL-6 expression. It is possible that parallel heme-induced pathways are activating IL-6 indirectly and with continuous hemolysis forming a feedback loop. With elevated cardiac PlGF at baseline in SCD mice and further inducibility by heme (343), cardiac hypertrophy may develop *via* IL-6 signaling (350). Therefore, it can be envisaged that prolonged hemolysis induced PlGF and IL-6 in SCD feeds the vicious cycle of inflammation *via* an autocrine feedback system resulting in reactivation of genetic cardiac hypertrophy program.

## Therapeutic Intervention in Hemolysis and Inflammation

The role of hemolysis and its attendant oxidant stress and inflammatory activation in SCD has been supported by the success of therapies that normalize these pathways. Hydroxyurea has pleiotropic effects that reduce hemolysis and offset its pathobiological consequences. The approval of hydroxyurea by the FDA in 1998 provided a watershed moment in the history of SCD (394, 395). Hydroxyurea treatment yielded an improved quality of life for SCD patients attributable to induction of fetal hemoglobin, slowing of chronic damage to several organs, including the brain (394–400). More than twenty years later, three new drugs; L-glutamine (Endari; reduction of pain-related hospital visit and length of stay) and crizanlizumab-tmca (Adakveo; reduction of frequency of VOC) and voxelotor (Oxbryta; inhibition of deoxygenated sickle hemoglobin polymerization), have been approved by the FDA for treatment of SCD (401). L-glutamine is thought to reverse the redox imbalance imposed by hemolysis and other sources of oxidative stress. Crizanlizumab blocks the inflammation-activated P-selection adhesive pathway. Voxelotor inhibits polymerization of sickle hemoglobin, with the most apparent effect of reduced hemolysis. Curative intent therapies have also shown evidence of reduced hemolysis. Although permanent cure afforded to patients through bone marrow transplant and gene therapy would be ideal, it would be quite expensive and the majority of patients with SCD live in areas lacking both economic and human resources needed to make these curative therapies broadly accessible (402). Importantly, the global majority of SCD patients live in resource-poor countries, with minimal access to these newer therapies and limited capacity for hematological monitoring requirements and other diagnostic equipment (1, 403). High childhood mortality rate ranging from 50–90% still prevail in these areas and acceptance of hydroxyurea as therapy is very low compared to developed countries (403–405).

Encouragingly, recent studies show the efficacy, safety and feasibility of using hydroxyurea treatment in children and adults with sickle cell anemia living in sub-Saharan Africa (406–408).

Clinical trials are underway to assess the potential of hemopexin intravenous infusion in the treatment of SCD (Clinicaltrials.gov identifier NCT04285827). In the Townes SCD mouse model, infusion of hemopexin reduced microvascular occlusion induced by hemoglobin infusion, hypoxia-reoxygenation, or lipopolysaccharide (83). Hemopexin mitigated induction of ICAM-1 and VCAM-1 *via* inhibition of NF-κB activation (83). In another study, treatment with Hpx attenuated free heme activation of complement pathways and kidney injury caused by complement deposition and inflammation in mice during hemolysis (157). Hemopexin also significantly decreased plasma heme concentration, pulmonary neutrophil extracellular trap (NET) formation, plasma DNA, neutrophil activation and NET-associated hypothermia in SCD mice (171).

## Conclusion

Hemolysis is a feature of many diseases, and in most cases occurring with acute and chronic inflammation that contributes to organ injury. Products of hemolysis activate several inflammatory pathways in many cell types, including cells in the innate immune system. Hemolysis appears to serve as a priming stimulus that combines with TLR4 signaling to a cascade of production of inflammatory cytokines which activate downstream pathophysiology. Therapeutic intervention targeting the upstream effects of hemolysis has potential to mitigate downstream innate immune system response and inflammation in treating patients with intravascular hemolytic disease.

## Author Contributions

All authors drafted the review. The first two authors contributed equally. GK approved the final version of this review. All authors contributed to the article and approved the submitted version.

## Funding

GK received support from NIH grants HL133864, MD009162 and from the Institute for Transfusion Medicine Hemostasis and Vascular Biology Research Institute at the University of Pittsburgh School of Medicine. OTG is supported by the American Society of Hematology Scholar Award.

## Conflict of Interest

GK is an employee of CSL Behring, LLC.

The remaining authors declare that the research was conducted in the absence of any commercial or financial relationships that could be construed as a potential conflict of interest.
